# How Do General Practitioners Conceptualise Advance Care Planning in Their Practice? A Qualitative Study

**DOI:** 10.1371/journal.pone.0153747

**Published:** 2016-04-20

**Authors:** Aline De Vleminck, Koen Pardon, Kim Beernaert, Dirk Houttekier, Robert Vander Stichele, Luc Deliens

**Affiliations:** 1 End-of-Life Care Research group, Ghent University & Vrije Universiteit Brussel (VUB), Brussels, Belgium; 2 Heymans Institute, Ghent University, Ghent, Belgium; 3 Department of Medical Oncology, Ghent University Hospital, Ghent, Belgium; Nathan Kline Institute and New York University School of Medicine, UNITED STATES

## Abstract

**Objectives:**

To explore how GPs conceptualise advance care planning (ACP), based on their experiences with ACP in their practice.

**Methods:**

Five focus groups were held with 36 GPs. Discussions were analysed using a constant comparative method.

**Results:**

Four overarching themes in the conceptualisations of ACP were discerned: (1) the organisation of professional care required to meet patients’ needs, (2) the process of preparing for death and discussing palliative care options, (3) the discussion of care goals and treatment decisions, (4) the completion of advance directives. Within these themes, ACP was both conceptualised in terms of content of ACP and/or in terms of tasks for the GP. A specific task that was mentioned throughout the discussion of the four different themes was (5) the task of actively initiating ACP by the GP versus passively waiting for patients’ initiation.

**Conclusions:**

This study illustrates that GPs have varying conceptualisations of ACP, of which some are more limited to specific aspects of ACP. A shared conceptualisation and agreement on the purpose and goals of ACP is needed to ensure successful implementation, as well as a systematic integration of ACP in routine practice that could lead to a better uptake of all the important elements of ACP.

## Introduction

Good quality communication between patients and health care professionals is essential in enabling patients with a life-limiting illness to understand their diagnosis, prognosis, treatment options and end-of-life care options in order to help them prepare and plan for their future care if they so desire [1]. Advance care planning (ACP) is a process of discussions with a patient about their wishes for future healthcare, in preparation for a time when they might lose capacity [2,3]. This process, involving healthcare professionals and family members or others, may be formally documented in an advance directive (AD) [4]. ACP is gaining attention worldwide for its perceived benefits in improving patient autonomy [5], concordance between patients’ preferences and end-of-life care received [6–8] and the quality of end-of-life care [9].

The traditional purpose of ACP has been to have patients prepare for incapacity and make treatment decisions in advance so that clinicians can attempt to provide care consistent with their goals at the end of life [10]. Emphasis was mainly put on the documentation of an agreement regarding medical care between the physician and patient through ADs. Yet, internationally their uptake remains limited [11,12]. Evidence also shows that there is no guarantee that these documents as such improve end-of-life care or correspond with future care preferences [13,14]. ADs are too narrowly focused on the patient’s right to refuse unwanted life sustaining treatment, they are often physically unavailable when needed, too vague to be useful in decision-making or at odds with the patient’s current clinical circumstances. In response to these concerns, there has been a shift from ACP as a static documentation of specific treatment preferences to an ongoing process of discussion and communication about a patient’s wishes [15]. It is expected that these changes in how ACP is operationalised can address these previously described concerns and increase the uptake and implementation of ACP [6].

In the Belgian health care system, as in many other countries, general practitioners (GPs) are core providers of medical care and the majority of people (95%) have a fixed GP with whom they have often built up a long-term relationship [16,17]. The GP is likely to have good clinical and contextual knowledge of each patient and is probably best placed to initiate an ACP discussion in a timely manner [18–20]. GPs play an important role in providing and coordinating end-of-life care in primary care, often in consultation with palliative home care teams [21,22]. Nonetheless, studies show that the practice of ACP in GPs remains limited [23–25]. It has been suggested before that differences in the conceptualisation of ACP have implications on how ACP is delivered and promoted in practice[15]. However, it remains unclear how ACP is conceptualised by GPs and how they perceive their role in it. Previous studies have focused mainly on the factors impeding GPs and patients in raising the topic of end-of-life care, and the main barriers hindering GPs in initiating ACP are the uncertainty about when to initiate ACP, fear of depriving patients from hope by initiating ACP, lack of time, lack of communication skills and discomfort with the process of ACP [26–29]. Some of the most important facilitators for GPs are when patients initiate ACP, education and training on ACP, and health care system changes that support the initiation of ACP such as sufficient time and financial compensation. However, the way in which GPs already engage in ACP today has not been studied and would be an important step towards improving the standard of practice by delineating how GPs currently understand and conceive ACP in their practice.

The aim of this qualitative study was to explore how GPs conceptualise ACP, by asking participants to describe their experiences with ACP in their practice. The goal of understanding the range of conceptualisations circulating among GPs is to provide new insights into how GPs can be helped to engage in the full and complex process of ACP.

## Methods

### Research design

In order to have an in-depth understanding of GPs’ perceptions and experiences regarding ACP, a qualitative study design was considered the most suitable. This study used the methodology of focus groups given the flexible approach it allowed for opens discussion and interaction between the participants [30]. The focus groups were conducted in Flanders, Belgium in 2012.

### Recruitment of participants

Participants in three focus groups were recruited by using existing peer-review groups for GPs, contacted via email. Nearly 97% of all full-time practicing GPs in Belgium are affiliated to such peer-review groups that are geographically determined, and where practice-related aspects are discussed four times per year [31]. Local peer-review groups consist of minimum 8 and maximum 25 members. Every GP who wants to be accredited in Belgium needs to be affiliated to one group and attendance at two out of four meetings per year is mandatory. All GPs who attended this meeting at the moment the focus groups were conducted also participated in the focus group. A fourth focus group was organised by contacting the coordinators of the palliative care networks in Flanders with the request to disseminate our invitation to participate in a focus group to the GPs active in palliative home care teams. Because of lower response, this focus group was complemented with other GPs recruited via the professional contacts of the network coordinators. A fifth focus group was organized with members from a large practice located in an urban region.

### Data collection

Each focus group was moderated and observed by two researchers (ADV, KP, RVS or LD) and lasted on average one and a half hours. At the start of each group the participants were informed that the discussions would be audiotaped, to which they all gave written informed consent. A topic guide, consisting of open questions and a set of prompts for each question, was used to generate the discussion (S1 Box). This topic guide was developed and reviewed within a multidisciplinary team of sociologists (ADV, DH, LD), psychologists (KP, KB) and one GP (RVS) and covered four themes: (1) experiences of GPs with ACP in their current practice, (2) attitudes regarding ACP, (3) perceived barriers to and facilitators for initiating ACP and (4) ways to improve initiation of ACP in general practice. A broad definition of ACP was introduced at the beginning of each focus group. ACP was defined as the voluntary process by which patients discuss their future treatment and end-of-life care preferences with their care providers, in case they lose capacity to make decisions or communicate their wishes for the future [3].

### Data analysis

The focus group discussions were transcribed verbatim. Analysis of the data was guided by a constant comparative method [32,33]. Firstly, ADV & KB independently read and openly coded two full focus group transcripts. The codes were discussed and mutually compared for similarities and differences until they could be grouped into categories related to the research questions. Subsequently, the five focus group transcripts were independently read and compared with the primary coding framework by all the members of the research team. Codes were added, modified or merged where necessary. ADV coded the remaining transcripts by applying the final coding framework, which was additionally checked by KB and KP for agreement on interpretation. An ongoing refinement of the coding framework resulted in overarching themes deduced from the categories. Finally, quotes were selected and approved by the research team to illustrate the results. The qualitative analysis software QSR NVIVO 10 was used for this research.

### Ethical aspects

Ethics approval for this study was given by the Commission of Medical Ethics of the University Hospital of Brussels (B.U.N. 143201212988).

## Results

Five focus groups were held with a total of 36 GPs (n = 9, n = 11, n = 4, n = 5, n = 7). The participants’ demographic characteristics are presented in Table 1.

**Table 1:** Characteristics of participating GPs (n = 36).

Characteristics	N
**Sex**	
Male	27
Female	9
**Age (years)**	
≤ 29	1
30–39	5
40–49	13
50–59	9
60–69	8
**Practice location**	
Urban	9
(Semi-) Rural	27
**Number of terminal patients in the last year**	
None	4
1–3	9
4–6	11
7–9	3
≥ 10	9
**Number of participants**	
Focus group 1	9
Focus group 2	11
Focus group 3	4
Focus group 4	5
Focus group 5	7

The GPs’ conceptualisation of ACP was explored by asking the participants to describe their experiences with ACP. At the beginning of each focus group, participants were asked whether they knew or were familiar with a definition of ACP. Although the GPs identified future (end-of-life) care conversations as an important aspect of general practice, many of them were not familiar with the specific term ‘ACP’. GPs with more experience in palliative care or GPs who received a formal training in palliative care were generally more familiar with the concept and process of ACP. However, once a general definition was introduced in the focus groups, most of the GPs could describe a range of experiences with these discussions but stated that they were mostly conducted in an informal way. After analysis, GPs’ experiences of ACP could be categorized in 5 overarching conceptualisations, either in terms of the content of the discussions and/or in terms of tasks for the GP. The conceptualisations of ACP could occur simultaneously in the narrative of a GP, however some GPs used only one conceptualisation to describe ACP in their practice.

### (1) Organisation of professional care to meet patients’ and families’ needs

Firstly, ACP was sometimes conceptualised as the process and discussions surrounding the organisation of professional care to meet patients’ and families’ needs, e.g. initiating (palliative) home care or moving to a nursing home. While some GPs only started to plan care in response to patients’ immediate or acute needs, others tried to prepare patients for future care decisions well in advance:

*“I prepare a lot of my older patients for the fact that one day that might have to move from their home to a nursing home*. *I know some people never think about the future or the fact that something might suddenly change*. *That is something I try to discuss at times*.*”*

(Male GP, 63 years, FG 3)

Planning care to address patients’ and families’ changing care needs often comprises collaboration between different informal and formal caregivers. A first important task in the organisational preparation for care is to establish a consensus between the patient’s preferences and the perspectives of the GP and of family members regarding adequate care (the initiation of palliative home care, moving to a nursing home, community nurse visits), which could sometimes require many discussions according to the GPs. The GPs indicated that it is not always an easy task to mediate between different perspectives about patients’ future care, especially if different distressed family members are involved.

*“It can be very difficult*. *For example*, *I am taking care of patient now that is really not doing well*. *Since a few days*, *he can’t get out of bed anymore*. *I tried to have a conversation with that man and his wife about the end of life*, *but on my way out his wife said to me*: *‘He does not like to talk about that*, *we are not going to do that anymore*.*’ So it is very difficult for me to organize palliative home care for them*. *They do not want to hear about it*.*”*

(Male GP, 44 years, FG 3)

The coordination of care often involves communication and collaboration with other health care professionals (e.g. specialists, community nurses or palliative home care teams). Although this communication and collaboration was not always judged as optimal, most GPs saw an important task for themselves in these contacts as an advocate for the patients:

*“A very important point for me*, *is the contact with the social services in the hospital*, *which does not happen not enough*. *You [the GP] have to make time for that and pick up the phone*. *That is important*.*”*

(Male GP, 65 years, FG 5)

### (2) Process of preparing for death: discussing palliative care options

Secondly, ACP was also conceptualised in terms of “bad news conversations”. The GPs construed ACP discussion then as the process of communicating a terminal diagnosis and bringing up the end-of-life to patients and their families. In this context, ACP was also described as the process of patients and their family preparing for death (psychologically, financially, making funeral arrangements, etc). Although many participants felt that it was also the treating specialist’s responsibility to explain the diagnosis and prognosis or discuss future treatment options with their patients, most GPs saw an important task in discussing palliative care options, comforting patients and reassuring terminal patients that they will be available and provide support until the end of life:

*“I believe that’s when you’ve got to tell them about what is available in palliative care*, *that they won’t be in any pain*, *that you’ll be there for them*, *that you’ll be able to*…*give them all the peace they need and a bit of assurance that you’ll walk that bit of road with them*.*”*

(Male GP, 42 years, FG 5)

“*Nowadays*, *they [the hospital] actually keep patients to themselves for a very long time*, *even at the point when nothing can be done for them anymore I think*. *Well*, *at certain wards this is definitely the case*. *They do that at the hospital for a really long time*, *even the moment they say*, *now we can’t do anything anymore*, *maybe you should go to the palliative unit now*, *while the patient actually won’t hear a lot about dying at home if we don’t come and explain it*, *I think*.*”*

(Female GP, 49 years, FG 1)

While some GPs considered the discussion of palliative care and the end-of-life as subjects that fit within a broader communication process about future care, others considered ACP as being synonymous with discussions only conducted at the end-of-life:

*“I think that during palliative care it is much discussed because the end is coming closer and the future is not that big anymore*, *so it is important to discuss it at that moment*.*”*

(Male GP, 35 years, FG 1)

### (3) Discussion of care goals and treatment decisions: hospital admissions and cancer treatments

When asked about specific care goals or treatment decisions that GPs discuss in advance with their patients, these topics were mostly mentioned during the focus groups: the reflection about and choices on different cancer treatments, and the experience of a previous acute hospital admission with linked to that patients’ preferences on a future admission if it should be required. However, the discussion of care goals and treatment decisions were mostly mentioned in reference to patients suffering from serious chronic or progressive illnesses such as cancer or organ failure, but never to patients with dementia.

*“I think we’ve got quite a lot of patients with dementia*, *but you won’t immediately start telling these people*: *soon you won’t know what you’re doing anymore*. *It’s time that you do something about it*, *that you start planning this*, *I think it’s a bit of a taboo to start discussing this*, *to tell someone with Alzheimer’s*, *in a year you won’t know what you’re doing anymore*.*”*

(Male GP, 44 years, FG 3)

### (4) The completion of advance directives

Fourthly, some participants talked specifically about the completion of documents regarding patient preferences in their conceptualisation of ACP. This was often in reference to the written plans about future care for their patients in nursing homes (either ADs or GP treatment orders) or the AD on euthanasia that was brought up by patients in their practice.

*“Something that we often see are ADs on euthanasia*, *when patients come by and say ‘look*, *this is in case if something happens to me*, *if I get a terminal illness’*. *They then want to sign a document in which they choose euthanasia and then don’t want to think about any more*. *That happens very often*.*”*

(Male GP, 45 years, FG 4)

The GPs explained that patients often have misunderstandings regarding the AD on euthanasia and it was considered an important task of the GP to inform their patients about ACP, the limitations of ADs, and ensure that ADs are valid and accurate.

*“Many patients say ‘if I am suffering dementia*, *I want euthanasia’*, *only there is no legal standing on that yet”*.

(Female GP, 48 years, FG 2)

It was noticeable that the documentation in general practice of patients’ wishes or other care preferences (e.g. the nomination of a substitute decision-maker) in the patients’ medical files was almost never mentioned during the focus groups.

### (5) The task of actively initiating vs. passively waiting to discuss ACP

Throughout the discussion of the four previous themes, it was noticed that the deliberation of actively initiating a discussion about these subjects versus passively waiting for patients’ initiation was a topic that was always discussed by the GPs in the focus groups. A number of participants considered actively initiating ACP discussions as an important task for GPs, but acknowledged that this often depends on the competencies and attitudes of the GP regarding ACP. Others were more reluctant to actively initiate ACP and commented that patients need to take initiative for these discussions, but it is the GP’s task at that moment to elaborate on the topic.

Being aware of trigger moments, listening to patient cues for initiating ACP, and making the right judgment of a patient’s willingness to discuss the topic were considered important skills for the initiation of ACP. However, a number of younger GPs indicated that experience with or expertise in ACP discussions is an important factor in actively initiating these discussions.

*“I think there are many different ways about it*, *as was said just before*. *Sometimes it’s because of a sudden diagnosis and you can talk to them [the patients] about it actively*. *Or sometimes you notice that patients try talks to you about it if you are a good listener*.*”*

(Female GP, 41 years, FG 1)

*“Also*, *many elderly people you visit quite often before they get ill and you know these people*, *so if you have a certain talent for it*, *there are many occasions to speak to them about it*.*”*

(Female GP, 60 years, FG 5)

However, it was also noted that not all GPs further explored patients’, sometimes vague, cues such as “*when needed*, *you [the GP] will help me*”. Especially in cases of terminally ill or very old patients, a few GPs stated to know their patients well enough to know it means not to prolong life unnecessary. However, most of the GPs responded to feel uncertain when patients expressed such vague wishes and preferred to explore these.

## Discussion and Conclusion

### Summary of main findings

Four overarching themes in the conceptualisations of ACP were discerned: (1) the organisation of professional care required to meet patients’ needs, (2) the process of preparing for death and discussing palliative care options, (3) the discussion of care goals and treatment decisions, (4) the completion of advance directives. Within these themes, ACP was both conceptualised in terms of content of ACP and/or in terms of tasks for the GP. A specific task that was mentioned throughout the discussion of the four different themes was the task of actively initiating ACP by the GP versus passively waiting for patients’ initiation.

### Strengths and limitations

To our knowledge, this is the first qualitative study to explore GPs’ conceptualisation of ACP on the basis of their experiences when engaging in ACP with their patients. The qualitative design, using different recruitment strategies [26], allowed us to gain insight into the complex range of views and experiences regarding ACP in daily practice from GPs with diverse backgrounds, experience and levels of interest in ACP. The multidisciplinary composition of the research team guaranteed interpretation of the data from a range of perspectives. However, the focus group composition may have presented a limitation. As a result of the different recruitment procedures for the focus groups, some participants (especially in the fourth and fifth focus group) might have had an increased interest in the topic. Also, most of the participant GPs were male (n = 27), so female GPs were underrepresented, as were GPs younger than 39 years (n = 6 vs. n = 30) although the national average age of GPs is 49 years in Belgium. Secondly, some focus groups were organised through existing peer-review groups for GPs, which might have presented some limitations. Although using such pre-existing groups has proven to be beneficial, as group discussions are naturally occurring during these meetings [34], it is also possible that some GPs might have feel restricted to report on their practice or experiences with ACP. However, using pre-existing groups offer the benefit of conducting focus groups with GPs that have no special or increased interest in ACP. Thirdly, although our study is aimed at theoretical rather than statistical generalization, the findings may not necessarily be generalizable to other countries were euthanasia is not legalized or countries that may have a very different context in which ACP is (partly) structurally embedded in national health care strategies [35].

### Comparison with existing literature

While ACP is suggested in the relevant literature as a comprehensive and multifaceted process of care planning that should be initiated at an early stage of a serious illness, the conceptualisations circulating among GPs tend to be more limited sometimes. While some GPs conceptualised ACP as a broad communication process in which many future care options such as future hospitalisations or the possibility of moving to a nursing home could be discussed, other viewed it specifically as a process that is to be initiated very late in the disease trajectory when death is imminent in order for patients and family to prepare for death. This suggests that ACP is only initiated by some GPs when end-of-life decisions need to be made, which entails rather an ‘ad hoc’ care planning instead of planning care in advance. However, the National Health Service in the UK makes a clear distinction in their National End of Life Care Programme between ‘general care planning’ on the one hand and ‘advance care planning’ on the other [36,37]. While general care planning focuses on patients’ current situation and making decisions about how to meet patients’ current needs in the context of available resources, ACP focuses on expressing preferences for directing future care decisions.

Based on the narratives about how they perform ACP in their own practice, the results of this study also suggest that the conceptualisation of ACP sometimes implies an engaging in specific aspects of ACP rather than engaging in multiple discussions that address all key elements as recommended in the literature [4,10,38–40]. These are ascertaining patients’ information and decision-making preferences, wishes for family involvement, understanding patients’ values, quality of life, fears and goals, explaining the nature and trajectory of serious illnesses, but also discussing disease outcomes, eliciting patients’ preferences for care and encouraging them to document their preferences and/or choose an appropriate surrogate decision maker. Some of these topics, such as the nomination of surrogate decision maker in advance or discussing preferences for care with dementia patients, were rarely mentioned during the focus groups and also the documentation of patients’ preferences did not occur often in general practice according to the GPs. Previous studies have shown that ACP is only documented in a small proportion of patients in general practice in Belgium, but verbally occurs more [23,24]. This finding is in line with the prevailing definition of ACP as a continuous process of communication that may not be narrowed to the formulation of a written AD. Nevertheless, to provide care in correspondence with the patient’s wishes, the documentaton of patients’ preferences may be very important when the decision making is urgent, without time for consultation, to use as a foundation for future discussions and the reviewing of preferences; or to ensure continuity of care because end-of-life care transitions occur rather frequently in Belgium [41,42].

Previous studies have argued that time constraints and the fact that GP consultations are mostly focused on a patient’s acute care needs restrict the possibilities of initiating these sensitive and time-consuming discussions [43,44]. However, implementing ACP as an iterative and multifaceted process of discussion throughout the illness course can help GPs in introducing difficult subjects gradually without having to find a single ‘perfect’ moment to introduce ACP. The results of this study also show that some GPs viewed ACP as a process undertaken by patients, while other viewed it as a their responsibility to initiate these discussions in order to gain a better understanding of patients’ wishes. It is noticeable that GPs consider experience as an important factor in actively initiating ACP. Communication skills can be learned and retained and do not reliably improve with experience alone. A previous study showed that a two hour educational programme about ACP specifically developed for GPs improved their confidence and ability to undertake ACP conversations with patients[38]. It includes active, practice-oriented strategies such as role-play exercises, feedback, the use of video role modelling, group discussions and feedback during the session as these are educational strategies which have shown to be most effective in improving communication skills. Offering GPs practical guidance on the content of ACP discussions might thus increase their use of it [45,46].

### Conclusion and implications for policy and practice

It has been recognized before that health care professionals have an important role in the promotion of ACP. They have a responsibility to initiate ACP in a timely way, by informing and educating patients [47]. However, our findings highlight significant differences in how ACP is conceptualised among GPs, which can cause confusion and conflict because the practice of individual GPs may vary according to how ACP is conceptualized, leading to reduced effectiveness of ACP [15]. Therefore, differences in the conceptualisation of ACP have important implications on how ACP should be promoted among GPs. A shared conceptualisation and agreement on the purpose and goals of ACP is needed to ensure successful implementation. Recently, the first guideline on ACP for health care professionals in Flanders was published, which can help in promoting a common view on ACP [48].

Nonetheless, previous studies have shown that the barriers to initiating ACP for GPs are complex and multifaceted [44,49]. It has been suggested that the combination of several interventions hold promise in implementing and systematizing the initiation of ACP with patients: education of physicians, systems to identify and trigger early discussions for eligible patients, patient and family education, structured formats to guide the discussions, dedicated sections in the electronic health record for recording information and continuous measurement [18,38].

Postponing ACP discussions until the end of life or waiting for patients to raise the subject may result in withholding from patients the right to receive information and plan their future care accordingly. This study illustrates that GPs have considerably varying conceptualisations of ACP in terms of the content of ACP discussions and in terms of tasks for the GP, of which some are more limited to only specific aspects of ACP. This can lead to confusion as the role of GPs may vary according to how ACP is conceptualised. A shared conceptualisation is needed to ensure a successful implementation of ACP.

## Supporting Information

S1 Box Topic guide of the focus groups with general practitioners. (DOCX)
