# Supplementary material for: How Do General Practitioners Conceptualise Advance Care Planning in Their Practice? A Qualitative Study
Source: PLoS One. 2016 Apr 20;11(4):e0153747. doi: 10.1371/journal.pone.0153747 (PMC4838248; doi:10.1371/journal.pone.0153747)
Supplement: S1 Box — (DOCX) [file pone.0153747.s001.docx]

Supporting Information File: Box 1 Topic guide of the focus groups with general practitioners

Introduction + Definition of ACP

**Theme 1: Experiences of GPs with ACP in practice**

1. Do you know the concept of ACP?

2. Do you have any experience with ACP in your practice?

**Theme 2: Attitudes regarding ACP**

1. Can you describe your attitude regarding ACP? Do you feel that ACP can be useful?

2. What are the main advantages and disadvantages of ACP for you?

**Theme 3: Perceived barriers and facilitators for initiating ACP**

1. Are there situations when the initiation of ACP is perceived as more difficult or easy for you?
2. Do you think that the (one of the three) specific disease trajectory of patients has an influence on the initiation of ACP for you?

**Theme 4: Interventions to improve the initiation of ACP in general practice**

1. What do you think could contribute to the improvement of initiating ACP for general practitioners?
